# Magnetohydrodynamic free convection between vertical parallel porous plates in the presence of induced magnetic field

**DOI:** 10.1186/s40064-015-1097-1

**Published:** 2015-07-08

**Authors:** Sarveshanand, A K Singh

**Affiliations:** DST-Centre for Interdisciplinary Mathematical Sciences, Banaras Hindu University, Varanasi, India; Department of Mathematics, Faculty of Science, Banaras Hindu University, Varanasi, India

**Keywords:** Porous plate, Free convection, Induced magnetic field, Induced current density

## Abstract

In this paper, the steady two-dimensional hydromagnetic free convective flow of an incompressible viscous and electrically conducting fluid between two parallel vertical porous plates has been considered. The effect of induced magnetic field arising due to the motion of an electrically conducting fluid is taken into account. The governing equations of the motion are a set of simultaneous ordinary differential equations and their analytical solutions in dimensionless form have been obtained for the velocity field, the induced magnetic field and the temperature field. The expression for the induced current density has been also obtained. The effects of various non-dimensional parameters on the velocity profile, the induced magnetic field profile, the temperature profile and the induced current density profile have been shown in the graphs. It is found that the effect of suction parameter is to decrease the velocity field and induced current density while it has increasing effect on the induced magnetic field.

## Background

Analysis on the free convection in an electrically conducting and viscous incompressible fluid in the presence of an external magnetic field has received considerable attention in the literature due to the useful applications in various branches of science and technology, such as fire engineering, nuclear science, combustion modeling, geophysics etc. The heat transfer and the skin-friction in such type of fluid motion can be reduced by applying a uniform external magnetic field. Depending on the complexity of the problem, the free convective studies have been carried out both theoretically and numerically. The first paper on the subject of hydromagnetic flows past a flat plate was presented by Rossow (1957). Denno (1972) investigated the effect of non uniform magnetic field on the magnetohydrodynamic channel flow between two parallel plates of infinite extent. Nanousis (1996) considered the two dimensional laminar flow of a viscous incompressible and electrically conducting fluid near an oscillating porous plate in the presence of uniform suction. Singha and Deka (2006), investigated the unsteady natural convection of an electrically conducting fluid between two heated parallel plates in the presence of a uniform magnetic field. The unsteady magnetohydrodynamic free convective flow and heat transfer along a vertical porous plate with variable suction and internal heat generation was discussed by Sharma and Singh (2008). Further, Palani and Srikanth (2009), analysed the hydromagnetic flow past a semi-infinite vertical plate with mass transfer. Free convective flow of heat generating/absorbing fluid between vertical porous plates with periodic heat input has been studied by Jha and Ajibade (2009).

Ellahi and Hameed (2012) have studied numerically the effects of nonlinear partial slip on the walls for steady flow and heat transfer of an incompressible, thermodynamically compatible third grade fluid in a channel. Series solutions of nonlinear partial differential equations with slip boundary conditions for non-Newtonian MHD flow in the porous space has been investigated by Zeeshan and Ellahi (2013). Sheikholeslami and Ganji (2014) have presented ferrohydrodynamic and magnetohydrodynamic effects on ferrofluid flow and convective heat transfer. MHD free convection in an electric semi-annulus field with nanofluid has been studied by Sheikholeslami et al. (2014). Effect of heat transfer on peristaltic motion of Oldroyd fluid in the presence of inclined magnetic field has been investigated by Khan et al. (2014). Sheikholeslami and Bandpy (2014) have presented free convection of ferrofluid in a cavity heated from below in the presence of an external magnetic field.

In the investigations concerned with the hydromagnetic free convective flows, the effect of induced magnetic field has been neglected in order to facilitate the mathematical analysis of the problem as simple. The induced magnetic field also generates its own magnetic field in the fluid and as a result of which it modifies the original magnetic field; at the same time their flow in the magnetic field produces mechanical forces which modify the motion of fluid. Therefore, in several physical situations it is required to include the effect of induced magnetic field in the hydromagnetic equations. Beg et al. (2009) have studied the non-similar, laminar, steady, electrically-conducting forced convection liquid metal boundary layer flow with the induced magnetic. A study on hydromagnetic free convective flow has been presented by Ghosh et al. (2010) by taking into account the effect of induced magnetic field. Further, Singh et al. (2010) have performed numerical study on the hydromagnetic free convective flow in the presence of an induced magnetic field. Kwanza and Balakiyema (2012) has investigated the hydromagnetic free convective flow past an infinite vertical porous plate with magnetic induction. Kumar and Singh (2013) have studied the unsteady magnetohydrodynamic free convective flow past a semi-infinite vertical wall by taking into account the induced magnetic field. Jha and Sani (2013) have presented the magnetohydrodynamic natural convective flow of an electrically conducting and viscous incompressible fluid in a vertical channel due to symmetric heating in the presence of induced magnetic field. Interaction of nano particles for the peristaltic flow in an asymmetric channel with the induced magnetic field has been studied by Akbar et al. (2014). Further, Akbar et al. (2015) have investigated the influence of induced magnetic field and heat flux with the suspension of carbon nanotubes for the peristaltic flow in a permeable channel.

In most of these studies, the boundaries are considered as the non-porous. The heat transfer and the skin-friction in such type of fluid motion can be reduced by considering the plates to be porous. Thus, in this paper, we have considered the hydromagnetic free convective flow of an electrically conducting and viscous incompressible fluid between parallel vertical porous plates with consideration of induced magnetic field. The governing equations corresponding to the velocity, induced magnetic and temperature fields have been solved analytically and further the expression for the induced current density have been also obtained. The effects of various parameters on the velocity, the induced magnetic field, the temperature and the induced current density profiles have been shown in the graphs.

## Governing equations

**Figure 1:**
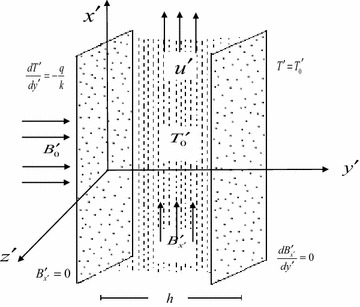
Physical model.

We consider the steady, free convective flow of an electrically conducting, viscous incompressible fluid between two infinite vertical porous plates with constant suction having suction velocity $V_{0}^{\prime }$. The $x^{\prime }$-axis is taken vertically upward along the plates and the $y^{\prime }$-axis normal to it as shown in the Figure 1. The distance between the plates is $h$. The one plate is kept at constant heat flux while the other is maintained at the constant temperature $T_{0}^{\prime }$. As the plates are of infinite extent, the variables describing the flow will depend only on the transverse coordinate $y^{\prime }$ and so the fluid velocity will have only one non zero component in the $x^{\prime }$-direction. A uniform magnetic field $\vec{B}_{0}^{\prime }$ of strength $B_{0}^{\prime }$ is applied perpendicular to the plates. The plate at $y^{\prime }$ = 0 is taken to be non-conducting while the other plate at y′ = h is taken to be electrically conducting. For a fluid with significant electrical conductivity $\sigma$, this in turn induces a magnetic field $B_{{x^{\prime}}}^{\prime }$ along the $x^{\prime }$-axis. Let $u^{\prime }$ be the velocity of the fluid along $x^{\prime }$-axis, then $\vec{v} = \left[ {u^{\prime } ,{\text{ V}}_{0}^{\prime } , \, 0} \right]$ is the velocity vector and $\vec{B} = \left[ {B_{{x^{\prime}}}^{\prime } ,{\text{ B}}_{0}^{\prime } , \, 0} \right]$ is the magnetic field vector of the considered problem.

The governing equations of the system is given by

1 $$\vartheta \frac{{d^{2} u^{\prime } }}{{dy^{\prime 2} }} + \frac{{\mu_{e} B_{0}^{\prime } }}{\rho }\frac{{dB_{{x^{\prime}}}^{\prime } }}{{dy^{\prime } }} + g\beta \left( {T^{\prime } - T_{0}^{\prime } } \right) + V_{0}^{\prime } \frac{{du^{\prime } }}{{dy^{\prime } }} = 0,$$

2 $$\frac{1}{{\sigma \mu_{e} }}\frac{{d^{2} B_{{x^{\prime}}}^{\prime } }}{{dy^{\prime 2} }} + B_{0}^{\prime } \frac{{du^{\prime } }}{{dy^{\prime } }} + V_{0}^{\prime } \frac{{dB_{{x^{\prime}}}^{\prime } }}{{dy^{\prime } }} = 0,$$

3 $$\frac{k}{{\rho C_{p} }}\frac{{d^{2} T^{\prime } }}{{dy^{\prime 2} }} + V_{0}^{\prime } \frac{{dT^{\prime } }}{{dy^{\prime } }} = 0,$$

with the boundary conditions

4 $$u^{\prime} = 0, \quad B^{\prime}_{{x^{\prime}}} = 0, \, \frac{{dT^{\prime}}}{{dy^{\prime}}} = - \frac{q}{k}\quad {\text{at}}\; y^{\prime} = 0,$$

5 $$u^{\prime} = 0, \, \frac{{dB^{\prime}_{{x^{\prime}}} }}{{dy^{\prime}}} = 0, \, T^{\prime} = T_{0}^{\prime } \quad{\text{at}}\; y^{\prime} = h.$$

Using the following non-dimensional parameters

6 $$y = \frac{{y^{\prime}}}{h}, \quad u = \frac{{\vartheta u^{\prime}}}{{g\beta h^{2} \varDelta T^{\prime}}}, \quad B = \frac{\vartheta }{{g\beta h^{2} \varDelta T^{\prime}}}\sqrt {\frac{{\mu_{e} }}{\rho }} B^{\prime}_{{x^{\prime}}} , \quad T = \frac{{T^{\prime} - T^{\prime}_{0} }}{{\varDelta T^{\prime}}}, \quad \varDelta T^{\prime} = \frac{hq}{k},\,\,\,\,\,\,\Pr = \frac{\mu Cp}{k}, \quad Pm = \vartheta \sigma \mu_{e} , \quad Ha = \frac{{B_{0} h}}{\vartheta }\sqrt {\frac{{\mu_{e} }}{\rho }} , \quad V_{0} = \frac{{V^{\prime}_{0} h}}{\vartheta },$$

the governing equations in non-dimensional form have taken the form

7 $$\frac{{d^{2} u}}{{dy^{2} }} + V_{0} \frac{du}{dy} + Ha\frac{dB}{dy} + T = 0,$$

8 $$\frac{{d^{2} B}}{{dy^{2} }} + V_{0} Pm\frac{dB}{dy} + HaPm\frac{du}{dy} = 0,$$

9 $$\frac{{d^{2} T}}{{dy^{2} }} + V_{0} \Pr \frac{dT}{dy} = 0,$$

with the boundary conditions in non dimensional form as

10 $$u = 0, \, B = 0, \, \frac{dT}{dy} = - 1\quad{\text{at}}\,\,y = 0,$$

11 $$u = 0, \, \frac{dB}{dy} = 0, \, T = 0 \quad {\text{at}}\,\,y = 1.$$

## Method of solution

Equations (7), (8), (9) are coupled system of ordinary differential equations with constant coefficients. This system of linear ordinary differential equations has been solved analytically by the theory of simultaneous ordinary differential equations. The expressions for the velocity field, the induced magnetic field and the temperature field in non-dimensional form are given by

12 $$u = \exp (K_{4} y)\left[ {K_{26} \cosh \left( {\sqrt {K_{5} } y} \right) + K_{27} \sinh \left( {\sqrt {K_{5} } y} \right)} \right] + K_{28} \exp (K_{1} y) + K_{29} y + K_{30} ,$$

13 $$B = \exp (K_{4} y)\left[ {K_{23} \cosh \left( {\sqrt {K_{5} } y} \right) + K_{22} \sinh \left( {\sqrt {K_{5} } y} \right)} \right] + K_{6} \exp (K_{1} y) + K_{7} y + K_{31,}$$

14 $$T = K_{2} \exp (K_{1} y) + K_{3} .$$

The induced current density is given by

15 $$J = - \frac{dB}{dy} = \exp (K_{4} y)\left[ {K_{32} \cosh \left( {\sqrt {K_{5} } y} \right) + K_{33} \sinh \left( {\sqrt {K_{5} } y} \right)} \right] - K_{1} K_{6} \exp (K_{1} y) - K_{7} .$$

The parameters $K_{1} , \, K_{2} , \ldots ,K_{31}$ used in the above equations are defined in the appendix.

## Results and discussion

The present magnetohydrodynamic free convection model is described by a number of physical parameters such as the Prandtl number $(\Pr )$, the magnetic Prandtl number $(Pm)$, the suction parameter $(V_{0} )$ and the Hartmann number $(Ha)$. The effects of various parameters on the velocity profile, the induced magnetic field profile and the induced current density profile are shown using the graphs. The parameters affecting the temperature distribution are the Prandtl number $(\Pr )$ and the suction parameter $(V_{0} )$ only and the effect of these parameters on the temperature profiles are also shown in the graphs.

**Figure 2:**
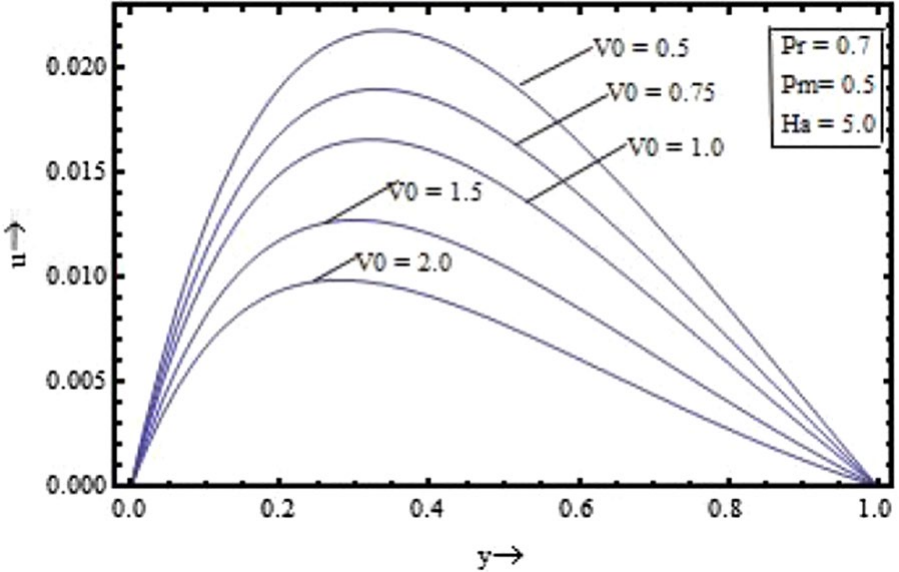
Variation of velocity with suction parameter $(V_{0} )$.

**Figure 3:**
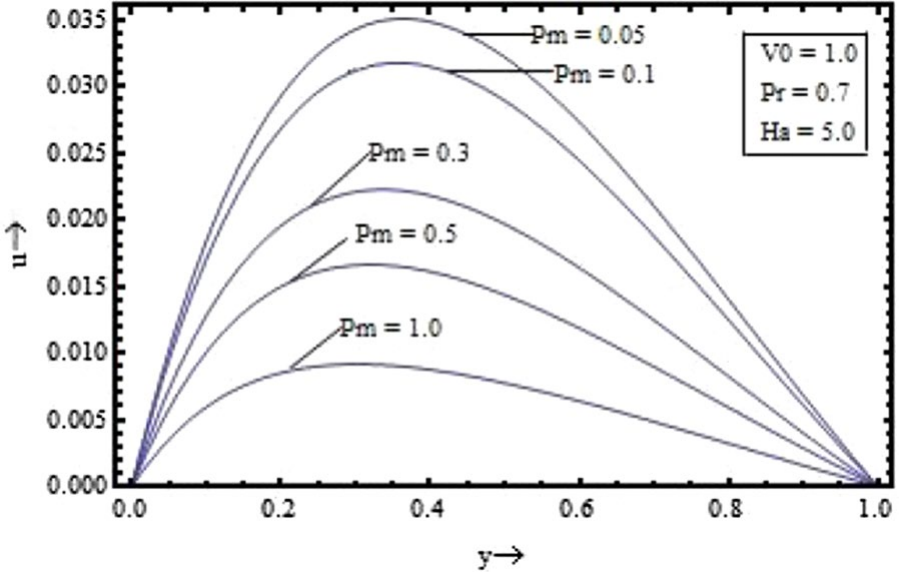
Variation of velocity with magnetic Prandtl number $(Pm)$.

**Figure 4:**
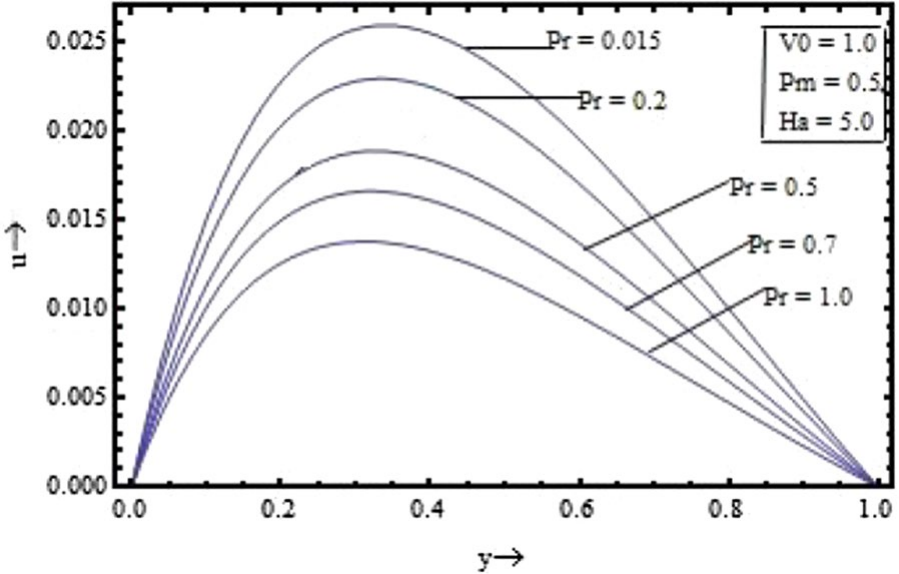
Variation of velocity with Prandtl number $(\Pr )$.

**Figure 5:**
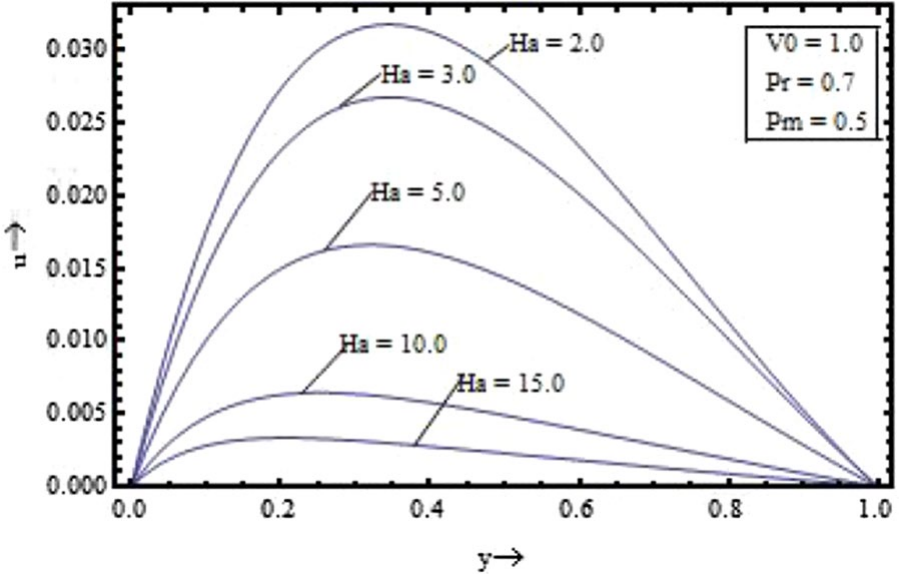
Variation of velocity with Hartmann number $(Ha)$.

Figures 2, 3, 4, 5 show the variation of the velocity with the parameters occurring in the governing equations. Figure 2 shows the effect of the suction parameter on the velocity distribution for $\Pr = 0.7, \, Pm \, = \, 0.5 \,$ and $Ha = 5$. It is found that the increase in the suction parameter leads to a decrease in the velocity profiles. Figure 3 shows the effect of the magnetic Prandtl number on the velocity profile for suction parameter $V_{0} = 1,$ the Prandtl number $\Pr = 0.7,$ and the Hartmann number $Ha = 5$. This figure clearly shows that the velocity of the fluid decreases as the magnetic Prandtl number increases. Figures 4 and 5 respectively show the variation of velocity with the Prandtl number $({\text{for }}V_{0} = 1, \, Pm = 0.5,{\text{ and }}Ha = 5)$ and the Hartmann number $({\text{for }}V_{0} = 1, \, Pm = 0.5,{\text{ and }}\Pr \, = 0.7)$.

It is seen that the velocity profile is found to decrease with the increase in the Prandtl number and the Hartmann number also. The velocity profiles are found to be almost parabolic type having their maximum value near the middle region but with increase in the magnetic Prandtl number and for large value of the Hartmann number its shape changes from the parabolic type to the flattered type. Thus, the fluid velocity can be reduced by application of strong external magnetic field.

**Figure 6:**
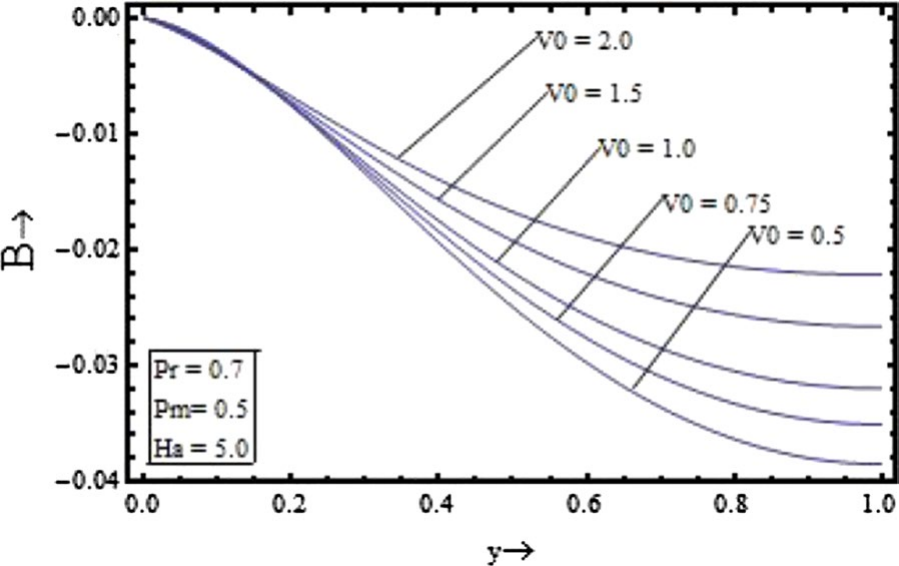
Variation of induced magnetic field with suction parameter $(V_{0} )$.

**Figure 7:**
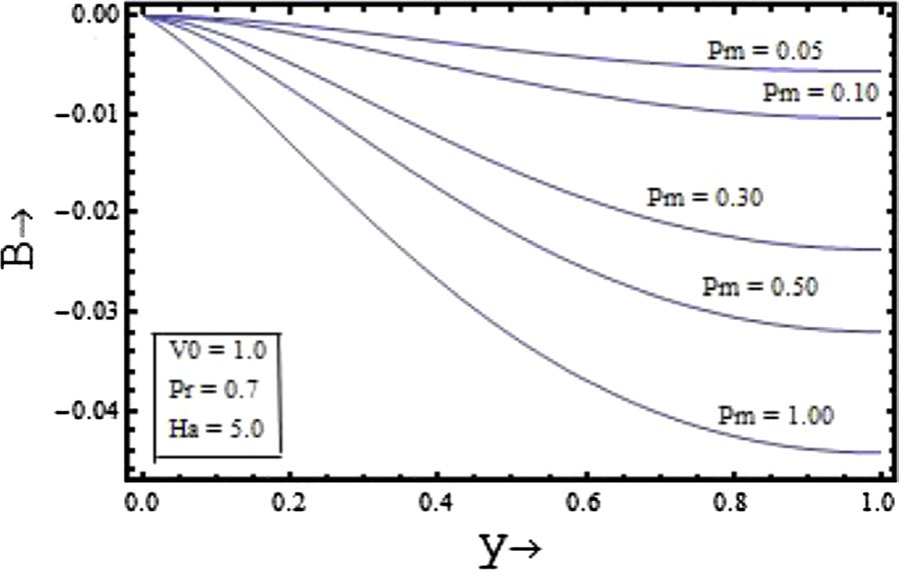
Variation of induced magnetic field with magnetic Prandtl number $(Pm)$.

**Figure 8:**
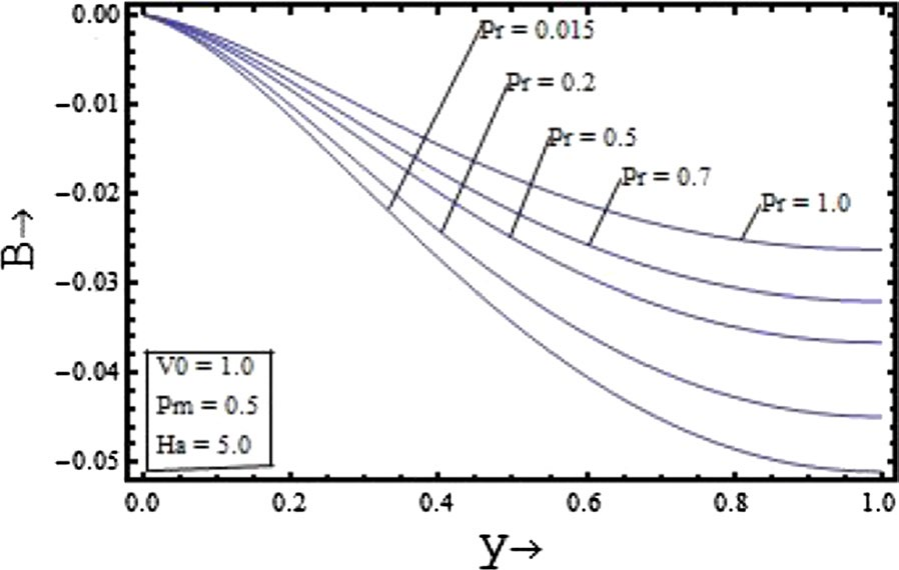
Variation of induced magnetic field with Prandtl number $(\Pr )$.

**Figure 9:**
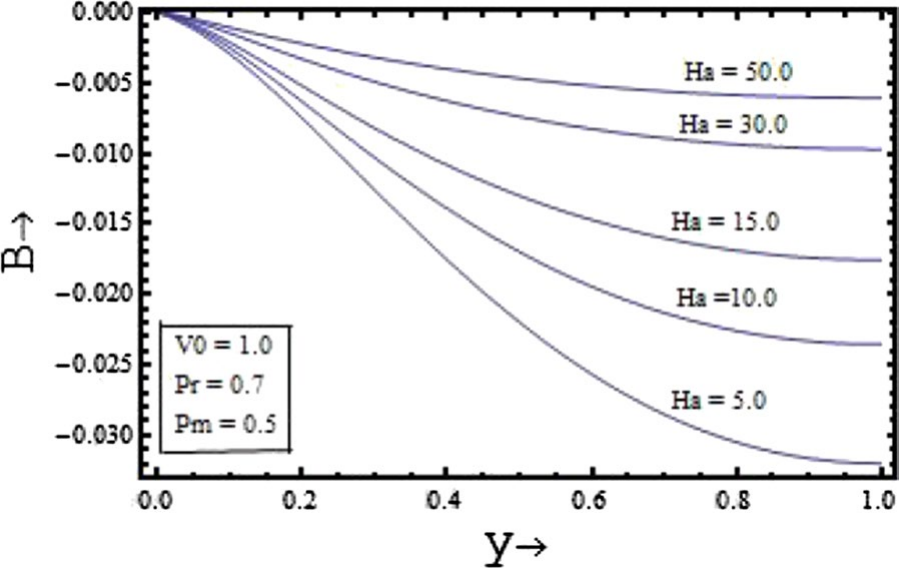
Variation of induced magnetic field with Hartmann number $(Ha)$.

Figures 6, 7, 8, 9 show the variation of induced magnetic field with the suction parameter, the Prandtl number, the Hartmann number and the magnetic Prandtl number. Figure 6 depicts the distribution of the induced magnetic with the suction parameter for $\Pr = 0.7, \, Pm = 0.5,{\text{ and }}Ha = 5$. It is observed that the induced magnetic field increases with increase in the suction parameter.

The induced magnetic field distribution plotted in Figure 7 with various values of $$Pm$$$$({\text{for }}\Pr = 0.7, \, V_{0} = 1,{\text{ and }}Ha = 5)$$, shows that the increase in the magnetic Prandtl number causes the decrease in the induced magnetic profile. In the Figures. 8 and 9 the profile of induced magnetic field shows the similar behavior of increasing with increase in the value of the Prandtl number $$\Pr$$$$({\text{for }}Pm = 0.5, \, V_{0} = 1,{\text{ and }}Ha = 5)$$ and with the increase in the value of the Hartmann number $$Ha$$$$({\text{for }}Pm = 0.5, \, V_{0} = 1,{\text{ and }}\Pr = 0.7)$$ respectively.

**Figure 10:**
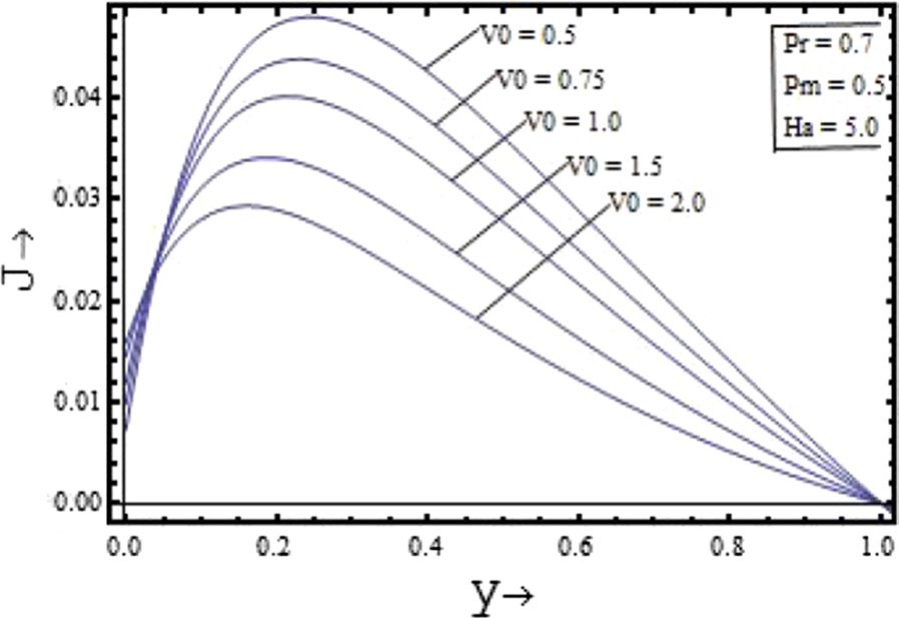
Variation of induced current density with suction parameter $(V_{0} )$.

**Figure 11:**
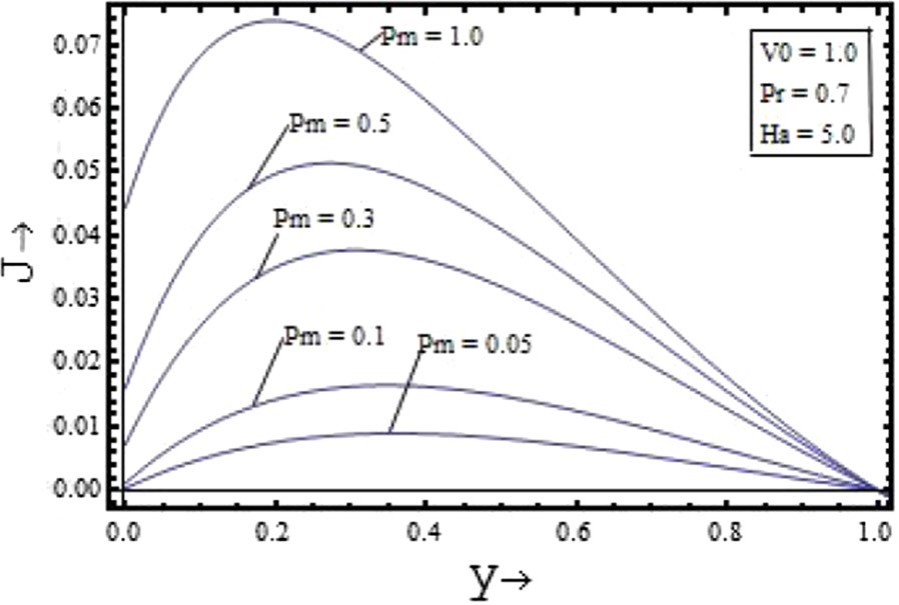
Variation of induced current density with magnetic Prandtl number $(Pm)$.

**Figure 12:**
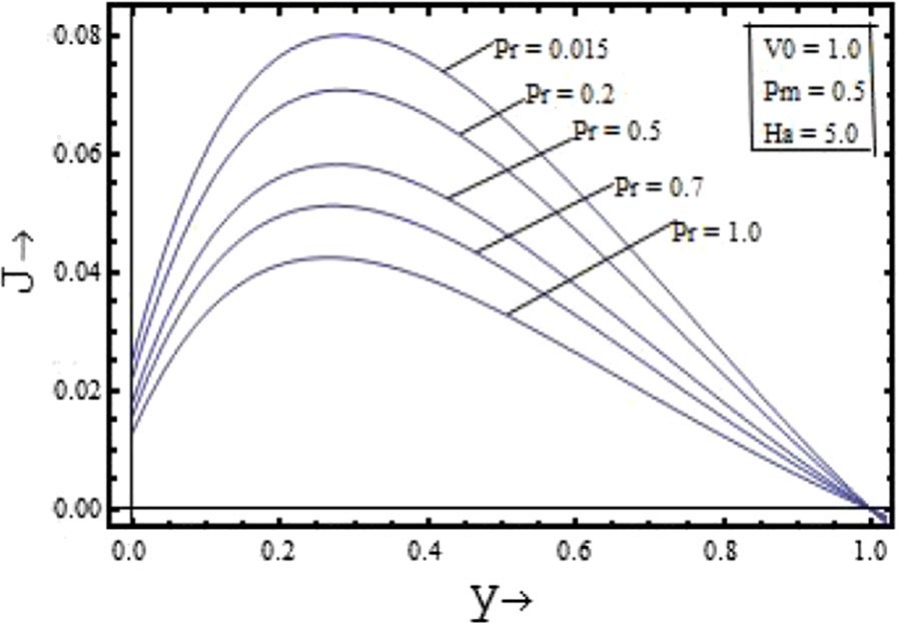
Variation of induced current density with Prandtl number $(\Pr )$.

**Figure 13:**
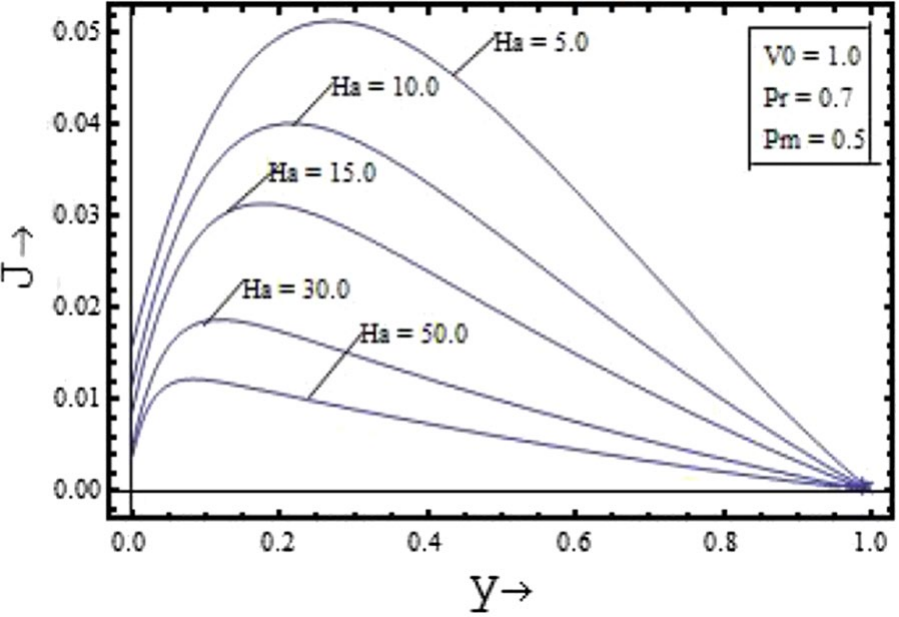
Variation of induced current density with Hartmann number $(Ha)$.

Figures 10, 11, 12, 13 illustrates the effect of the parameters $$V_{0} , \, Pm,{\text{ Pr and }}Ha$$ on the induced current density profiles respectively. Figure 10 presenting the variation of induced current density with the suction parameter $$V_{0} \, ({\text{ for }}\Pr = 0.7, \, Pm = 0.5,{\text{ and }}Ha = 5)$$, shows that the induced current density profile decreases with the increase in the value of suction parameter. The variation of the induced current density with the Prandtl number $$\Pr$$$$({\text{for }}Pm = 0.5, \, V_{0} = 1,{\text{ and }}Ha = 5)$$, is shown in the Figure 12 and its variation with the Hartmann number $$Ha$$$$({\text{for }}Pm = 0.5, \, V_{0} = 1,{\text{ and }}\Pr = 0.7)$$, is shown in the Figure 13. It is clear from the graphs that effect of the Prandtl number and the Hartmann number on the induced current density profile is found to have a decreasing nature. The induced current density increases with the increase in the value of the magnetic Prandtl number $$Pm$$$$({\text{for }}\Pr = 0.7, \, V_{0} = 1,{\text{ and }}Ha = 5)$$ as shown in the Figure 11.

**Figure 14:**
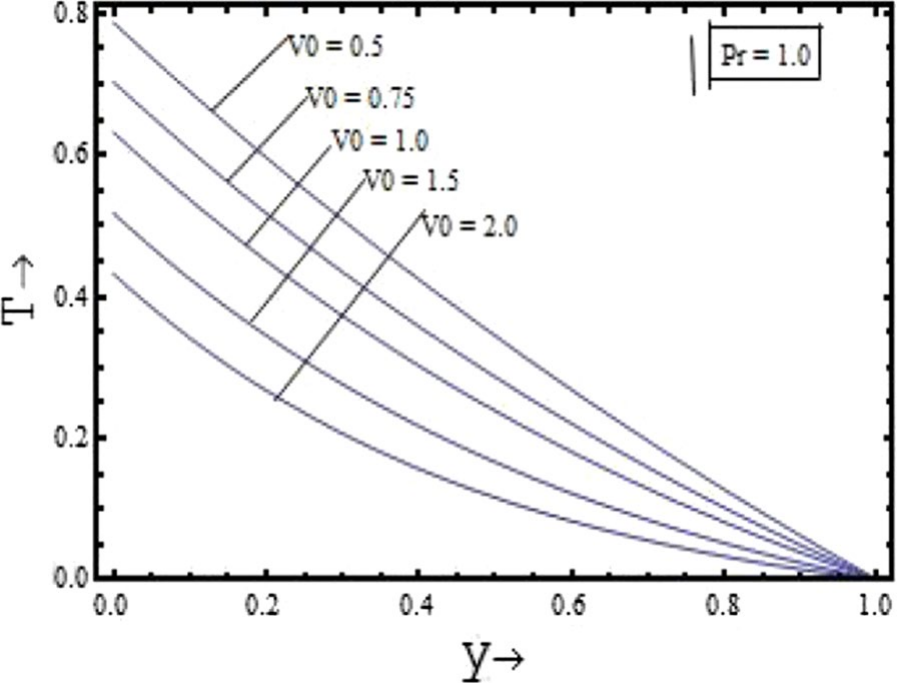
Variation of temperature field with suction parameter $(V_{0} )$.

**Figure 15:**
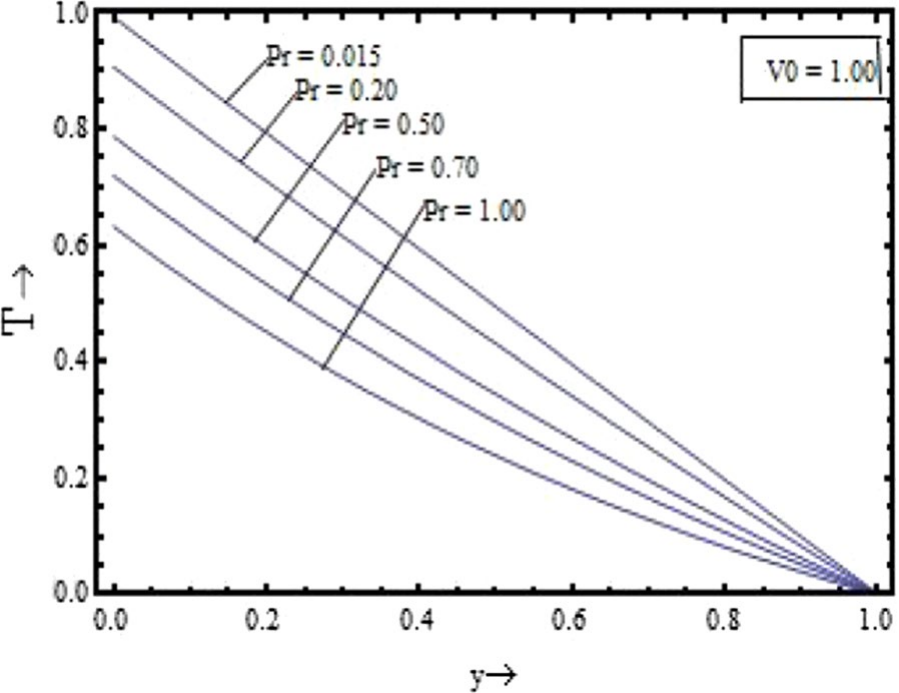
Variation of temperature field with Prandtl number $(\Pr )$.

The temperature variation are shown in the Figure 14 with various values of the suction parameter $(V_{0} )$ at $\Pr = 1.0$ and in the Figure 15 with different values of the Prandtl number $(\Pr )$ at $V_{0} = 1.0$. The temperature field distribution is found to have the decreasing nature with increase in the both parameters.

Knowing the expression of the velocity, the other physical quantity of interest is the skin friction. Using the Eq. (12), the skin friction on both the walls in non-dimensional form are given by

16 $$\tau_{0} = \left( \frac{du}{dy} \right)_{y = 0} = K_{1} K_{23} + K_{4} K_{26} + K_{27} \sqrt {K_{5} } + K_{29} ,$$

17 $$\tau_{1} = - \left( \frac{du}{dy} \right)_{y = 1} = e^{{K_{4} }} \left[ {\left( {K_{4} K_{26} + K_{27} \sqrt {K_{5} } } \right)\cosh \sqrt {K_{5} } + \left( {K_{4} K_{27} + K_{26} \sqrt {K_{5} } } \right)\sinh \sqrt {K_{5} } } \right] + K_{1} K_{23} e^{{K_{1} }} + K_{29} .$$

The effects of suction parameter and the magnetic Prandtl number on the skin friction on the two plates are shown in the Table 1. This table clearly shows the skin friction on both the plates decreases with increase in the value of the suction velocity. Further, with increase in the value of the magnetic Prandtl number, the skin friction decreases on the plate at $y = 0$ while increases on the other plate at $y = 1$. The effects of the Prandtl number and the Hartmann number on the skin friction are shown in the Table 2. It is seen that as the value of the Prandtl number increases the skin friction decreases on both the plates while with increase in the value of the Hartmann number, the skin friction decreases on the plate at $y = 0$ and increases on the plate at $y = 1$.

**Table 1:** Effect of suction velocity and magnetic Prandtl number on skin friction

$V_{0}$	$\Pr = 0.7, \, Pm = 0.5, \, Ha = 5.0$		$Pm$	$V_{0} = 1.0,{ \Pr } = 0.7, \, Ha = 5.0$	
	$\tau_{0}$	$\tau_{1}$		$\tau_{0}$	$\tau_{1}$
0.50	0.201282	0.016061	0.05	0.942302	−0.288576
0.75	0.189208	0.010222	0.10	0.579872	−0.123172
1.00	0.177648	0.006543	0.30	0.268411	−0.011856
1.50	0.156469	0.002602	0.50	0.177648	0.006543
2.00	0.138151	0.000717	1.00	0.078089	0.0182087

**Table 2:** Effect of Prandtl number and Hartmann number on skin friction

$\Pr$	$V_{0} = 1.0, \, Pm = 0.5, \, Ha = 5.0$		$Ha$	$V_{0} = 1.0, \, Pm = 0.5, \, \Pr = 0.7$	
	$\tau_{0}$	$\tau_{1}$		$\tau_{0}$	$\tau_{1}$
0.015	2.49099	2.69599	2.00	0.496807	−0.088505
0.20	−0.062465	0.135359	3.00	0.304076	−0.009525
0.50	−0.165730	0.023090	5.00	0.177665	0.006543
0.70	−0.177648	0.006543	10.0	0.085344	0.003380
1.00	−0.181191	−0.002208	15.0	0.055678	0.001650

## Conclusion

The hydromagnetic free convective flow between two vertical parallel porous plates has been studied by taking into account the effect of induced magnetic field. It is observed that the increase in the suction parameter, the Prandtl number, the magnetic Prandtl number and the Hartmann number leads to a decrease in the velocity profiles. It is also observed that with increase in the suction parameter, the Prandtl number and the Hartmann number the induced magnetic field increases while decreases with increase in the magnetic Prandtl number. The induced current density profile increases with increase in the magnetic Prandtl number while it decreases with increase in the suction parameter, the Hartmann number and the Prandtl number. The velocity and induced magnetic field can be controlled by adjusting suction/injection velocity on the porous plates while making engineering designs.

## Appendix

$$K_{1} = - V_{0} \Pr , \quad K_{2} = - \frac{1}{{K_{1} }}, \quad K_{3} = \frac{{\exp (K_{1} )}}{{K_{1} }}, \quad K_{4} = - \frac{{V_{0} (1 + Pm)}}{2},$$

$$\quad K_{5} = \frac{{V_{0}^{2} (1 - Pm)^{2} + 4PmHa^{2} }}{4}, \quad K_{6} = \frac{{HaPmK_{2} }}{{K_{1} \left\{ {K_{1}^{2} + V_{0} K_{1} (1 + Pm) + Pm(V_{0}^{2} - Ha^{2} )} \right\}}},$$

$$\, K_{7} = \frac{{HaK_{3} }}{{(V_{0}^{2} - Ha^{2} )}}, \quad K_{8} = \frac{{V_{0} }}{{Pm(V_{0}^{2} - Ha^{2} )}}, \quad K_{9} = - \frac{Ha}{{(V_{0}^{2} - Ha^{2} )}},$$

$$\, K_{10} = - \frac{{V_{0} HaK_{3} (1 + Pm)}}{{Pm(V_{0}^{2} - Ha^{2} )^{2} }}, \quad K_{11} = V_{0} PmK_{9} , \quad K_{12} = (V_{0} PmK_{8} - 1),$$

$$K_{13} = \left( {V_{0} Pm + K_{4} } \right), \quad K_{14} = - \left[ {\left( {K_{1} + V_{0} Pm} \right)K_{6} + V_{0} PmK_{10} + K_{7} } \right],$$

$$K_{15} = \exp (K_{4} )\left[ {\left( {V_{0} Pm + K_{4} } \right)\cosh (\sqrt {K_{5} } ) + \sqrt {K_{5} } \sinh (\sqrt {K_{5} } )} \right],$$

$$K_{16} = \exp (K_{4} )\left[ {\left( {V_{0} Pm + K_{4} } \right)\sinh (\sqrt {K_{5} } ) + \sqrt {K_{5} } \cosh (\sqrt {K_{5} } )} \right],$$

$$K_{17} = - \left[ {\left( {V_{0} Pm + K_{1} } \right)K_{6} \exp (K_{1} ) + \left( {V_{0} Pm + 1} \right)K_{7} + V_{0} PmK_{10} } \right],$$

$$K_{18} = - (K_{6} + K_{10} ), \quad K_{19} = \exp (K_{4} )\left[ {K_{4} \cosh (\sqrt {K_{5} } ) + \sqrt {K_{5} } \sinh (\sqrt {K_{5} } )} \right],$$

$$K_{20} = \exp (K_{4} )\left[ {K_{4} \sinh (\sqrt {K_{5} } ) + \sqrt {K_{5} } \cosh (\sqrt {K_{5} } )} \right], \quad K_{21} = - \left[ {K_{1} K_{6} \exp (K_{1} ) + K_{7} } \right],$$

$$K_{22} = \frac{{K_{19} (K_{14} - K_{17} ) - K_{21} (K_{13} - K_{15} )}}{{K_{19} (\sqrt {K_{5} } - K_{16} ) - K_{20} (K_{13} - K_{15} )}}, \quad K_{23} = \frac{{K_{14} - K_{17} - K_{22} (\sqrt {K_{5} } - K_{16} )}}{{(K_{13} - K_{15} )}},$$

$$K_{24} = \frac{{K_{9} K_{11} - K_{11} K_{18} - K_{23} (K_{9} K_{13} - K_{11} ) - K_{9} K_{22} \sqrt {K_{5} } }}{{K_{9} K_{12} - K_{8} K_{11} }}, \, K_{25} = \frac{{K_{14} - K_{12} K_{24} - K_{13} K_{23} - K_{22} \sqrt {K_{5} } }}{{K_{11} }},$$

$$K_{26} = - \frac{{\left( {V_{0} PmK_{23} + K_{4} K_{23} + K_{22} \sqrt {K_{5} } } \right)}}{HaPm}, \quad K_{27} = - \frac{{\left( {V_{0} PmK_{22} + K_{4} K_{22} + K_{23} \sqrt {K_{5} } } \right)}}{HaPm},$$

$$K_{28} = - \frac{{(K_{1} + V_{0} Pm)K_{6} }}{HaPm}, \quad K_{29} = - \frac{{V_{0} K_{7} }}{Ha}, \quad K_{30} = \frac{{K_{24} - K_{7} - V_{0} PmK_{10} - V_{0} PmK_{8} K_{24} - V_{0} PmK_{9} K_{25} }}{HaPm},$$

$$K_{31} = K_{8} K_{24} + K_{9} K_{25} + K_{10,} \quad K_{32} = - (K_{4} K_{23} + K_{22} \sqrt {K_{5} } ), \quad K_{33} = - (K_{4} K_{22} + K_{23} \sqrt {K_{5} } ).$$

## List of symbols

$u^{\prime }$: velocity of the fluid along the $x^{\prime }$-direction
$u$: velocity of the fluid along the $x^{\prime }$-direction in non-dimensional form
$$\vec{V}^{\prime }$$: velocity vector
$$V_{0}^{\prime }$$: suction velocity
$$V_{0}$$: Suction parameter
$\vec{B}^{\prime }$: Magnetic field vector
$B_{0}^{\prime }$: Applied external magnetic field
$B_{{x^{\prime } }}^{\prime }$: Induced magnetic field in $x^{\prime }$-direction
$B$: Non-dimensional induced magnetic field in $x^{\prime }$-direction
$$Ha$$: Hartmann number
$T_{0}^{\prime }$: Initial temperature of the fluid
$T^{\prime }$: Temperature of the fluid
$T$: Temperature of the fluid in non-dimensional form
$g$: Acceleration due to gravity
$$h$$: Characteristic length
$$k$$: Thermal conductivity
$$q$$: Heat flux
$J$: Induced current density
$$\Pr$$: Prandtl number
$Pm$: Magnetic Prandtl number
$C_{p}$: Specific heat at constant pressure
$\beta$: Coefficient of thermal expansion
$\mu$: Coefficient of viscosity
$$\mu_{e}$$: Magnetic permeability
$$\eta$$: Magnetic diffusivity
$\vartheta$: Kinematic viscosity of the fluid
$\rho$: Density of the fluid
$\sigma$: Conductivity of fluid
